# Remnant Cholesterol Inflammatory Index and Its Association With All‐Cause Mortality Among General Population and Individuals With Cardiovascular–Kidney–Metabolic Syndrome Stages 0–3: Evidence From Two Nationwide Studies

**DOI:** 10.1002/clc.70297

**Published:** 2026-04-13

**Authors:** Chen‐Zhang Ou, Zhong‐Qiang Liu, Yu‐Jun Xiong, Shiqin Chen, Tian Lv, Jingjing Lou

**Affiliations:** ^1^ Department of Urology Jiangnan University Medical Center, Wuxi No.2 People's hospital Wuxi Jiangsu China; ^2^ Department of Obstetrics and Gynecology Xinyang Central Hospital Xinyang Henan China; ^3^ Department of Gastroenterology Beijing Hospital, National Center of Gerontology, Institute of Geriatric Medicine, Chinese Academy of Medical Sciences Beijing P.R. China; ^4^ Department of Neurology Yuhuan Second People's Hospital Yuhuan China; ^5^ Department of Neurology Zhuji Affiliated Hospital of Wenzhou Medical University Zhuji China

**Keywords:** all‐cause mortality, cardiovascular–kidney–metabolic syndrome, metabolic parameters, RCII

## Abstract

**Background:**

Early identification of mortality risk in cardiovascular–kidney–metabolic (CKM) stages 0–3 remains challenging. The remnant cholesterol inflammatory index (RCII) has been associated with adverse outcomes, but its comparative prognostic performance in early CKM stages is unclear.

**Methods:**

We analyzed data from two nationally representative cohorts, the U.S. National Health and Nutrition Examination Survey (NHANES) and the China Health and Retirement Longitudinal Study (CHARLS). RCII was compared with established metabolic indices for all‐cause mortality using Cox regression, receiver operating characteristic analyses, and restricted cubic splines, with stratification by CKM stages.

**Results:**

Across both cohorts, higher RCII was consistently associated with increased all‐cause mortality in the general population and among individuals with CKM stages 0–3. RCII demonstrated superior discriminatory performance compared with other metabolic indices. These associations remained robust after multivariable adjustment and were driven primarily by individuals in the highest RCII category. In NHANES, RCII showed comparable associations with cardiovascular and non‐cardiovascular mortality.

**Conclusion:**

RCII is a strong and independent predictor of all‐cause mortality across diverse populations and CKM stages 0–3.

## Graphical abstract

RCII consistently outperformed other markers and exhibited a strong, near‐linear association with mortality risk across both general populations and those with CKM stages 0–3.

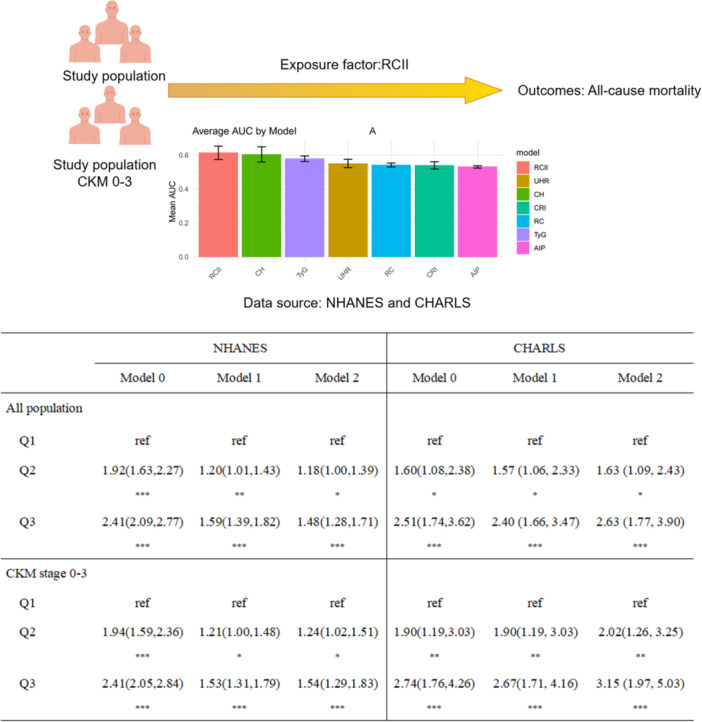

## 1 Introduction

Cardiometabolic biomarkers have emerged as powerful predictors of mortality risk, yet their comparative prognostic value across different disease stages remains poorly understood [1]. A growing body of evidence suggests that cardiometabolic risk is driven by the convergence of dyslipidemia, insulin resistance, and chronic low‐grade inflammation. To operationalize these complex processes, several composite metabolic indices have been proposed, each capturing different dimensions of cardiometabolic dysfunction. Some indices primarily reflect lipid‐related atherogenic burden, whereas others emphasize inflammatory activity or glucose–lipid interactions. However, whether these markers provide comparable prognostic information, particularly at early stages of cardiovascular–kidney–metabolic impairment, remains unclear [2, 3, 4, 5, 6, 7, 8]. These parameters reflect distinct yet interconnected pathophysiological pathways, including lipid metabolism dysregulation, insulin resistance, and chronic inflammation [9]. However, their relative importance for mortality prediction, particularly in early‐stage cardiovascular‐kidney‐metabolic (CKM) syndrome, has not been systematically evaluated.

While the CKM staging framework has advanced the conceptual integration of metabolic, cardiovascular, and renal disease, current stage definitions rely largely on categorical clinical thresholds. As a result, individuals with ongoing subclinical metabolic–inflammatory injury may not be adequately distinguished within early CKM stages. Identifying biomarkers that reflect continuous risk across CKM stage 0–3 therefore remains a critical unmet need [10, 11, 12]. This represents a critical gap in preventive cardiology, as accurate risk stratification in these early stages could enable more targeted interventions to prevent disease progression.

Previous studies have explored the individual prognostic relevance of these cardiometabolic markers in various clinical settings. Remnant cholesterol inflammatory index (RCII), which integrates lipid‐related atherogenesis and systemic inflammation, has been linked to increased cardiovascular mortality and adverse metabolic outcomes in large cohort analyses [13]. The ratio of hs‐CRP to HDL‐C, often denoted as CH, serves as a valuable composite marker reflecting both inflammation and lipid metabolism. This ratio has been linked to atherosclerotic burden and the severity of metabolic syndrome, with dysregulated hepatic cholesterol flux potentially playing a key role [14, 15]. Uric acid to HDL‐cholesterol ratio (UHR), a composite measure reflecting oxidative stress and impaired reverse cholesterol transport, has emerged as a strong predictor of both all‐cause mortality and myocardial infarction [16, 17]. Similarly, Castelli's Risk Index‐II (CRI), calculated as the ratio of LDL to HDL‐C, has long been associated with increased cardiovascular risk and subclinical atherosclerosis [18]. Remnant cholesterol (RC), representing cholesterol‐rich triglyceride remnants, has been independently associated with incident cardiovascular events and residual inflammatory risk, particularly among statin‐treated individuals [19]. Triglyceride‐glucose index (TyG) index, a surrogate for insulin resistance, has been linked to incident diabetes, coronary artery disease, and overall mortality [20]. Lastly, atherogenic index of plasma (AIP), defined as the logarithmic ratio of triglycerides to HDL‐C, has been shown to predict metabolic syndrome, arterial stiffness, and long‐term cardiovascular events across multiple populations [21]. Multiple lipid‐ and inflammation‐related markers have been proposed to explain residual cardiometabolic risk beyond traditional LDL‐C reduction. However, these markers are typically evaluated in isolation, despite substantial biological overlap between dyslipidemia and chronic inflammation. In the present study, we approached this issue from a comparative perspective by evaluating the prognostic performance of several established metabolic indices simultaneously. This framework allows RCII to be interpreted not merely as a novel construct, but as a composite signal that integrates lipid‐related and inflammatory risk dimensions that appear to converge early in the CKM continuum. Moreover, limited evidence exists regarding their combined performance in diverse populations such as those represented in NHANES and CHARLS. A systematic assessment of their relative strengths may help clarify which biomarkers are most informative at specific disease stages, thereby improving early risk detection and management strategies [22].

To address these knowledge gaps, we conducted a comparative analysis of seven key metabolic parameters (RCII, CH, UHR, CRI, RC, TyG, and AIP) using data from the National Health and Nutrition Examination Survey (NHANES) and the China Health and Retirement Longitudinal Study (CHARLS). Through ROC curve analysis, we systematically evaluated their predictive performance for all‐cause mortality in both general populations and across CKM stages 0–3. Importantly, rather than merely confirming previously reported associations, this study aims to systematically compare RCII with multiple established metabolic indices within the same analytical framework, and to determine whether RCII provides incremental and stage‐specific prognostic information in individuals with early CKM stages (0–3).

## 2 Materials and Methods

### 2.1 Study Design and Participants

This study leveraged data from two large‐scale, nationally representative cohorts: the National Health and Nutrition Examination Survey (NHANES) in the United States and the China Health and Retirement Longitudinal Study (CHARLS). NHANES adopts a complex, multistage, stratified probability sampling design to obtain a representative sample of the non‐institutionalized U.S. population. Its primary objective is to assess the health and nutritional status of individuals across the country [23]. The survey protocol is approved by the Institutional Review Board of the National Center for Health Statistics, and all participants provide written informed consent prior to data collection. NHANES gathers extensive health‐related information through standardized procedures, encompassing demographic characteristics, dietary intake, physical examinations, laboratory analyses, and responses to structured questionnaires [24]. CHARLS is a longitudinal study of Chinese adults aged 45 years and older, designed to represent the national population (http://charls.pku.edu.cn/). The baseline sample was drawn from 450 communities across 150 counties or districts in 28 provinces, with follow‐up waves conducted from 2011 to 2020 [25].

**FIGURE 1:**
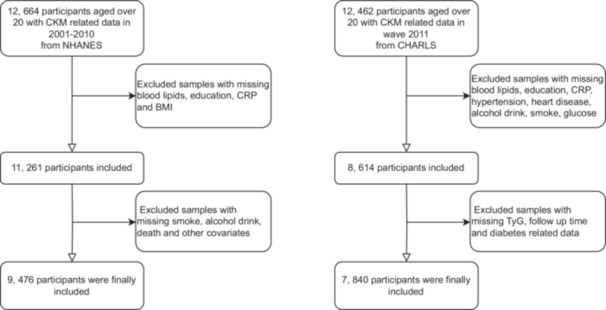
Flowchart of participant screening in NHANES and CHARLS.

During the 2001–2009 NHANES cycles, a total of 12 664 participants with available CKM‐related data were initially considered. Individuals with missing information on blood lipids, education level, C‐reactive protein (CRP), body mass index (BMI), smoking status, alcohol consumption, mortality, or other key covariates were excluded from the analysis. For the CHARLS cohort, data from the baseline wave in 2011 were utilized, with follow‐up through 2020. The 2011 wave included 12 462 respondents, from whom those lacking data on blood lipids, education, hsCRP, hypertension, heart disease, glucose, smoking, alcohol use, or other relevant variables were excluded. These exclusion criteria were applied to ensure data completeness and enhance the validity and reliability of the statistical analyses (Figure 1).

### 2.2 Definition of CKM Syndrome Across Stages 0–3

CKM syndrome is categorized into Stages 0–3 according to the 2023 AHA Presidential Advisory [26]. Stage 0 indicates the absence of cardiometabolic and renal risk factors, including normal body size, metabolic parameters, kidney function, and no signs of CVD. Stage 1 involves abdominal obesity and/or prediabetes, reflecting early adipose tissue dysfunction. Stage 2 includes individuals with established metabolic conditions such as type 2 diabetes, hypertension, hypertriglyceridemia, or moderate‐to‐high risk CKD (Stage G3). Stage 3 is characterized by subclinical CVD in individuals with obesity, metabolic abnormalities, or CKD, defined by advanced CKD (eGFR < 30 mL/min/1.73 m^2^) or a 10‐year CVD risk ≥ 20% based on the Framingham Risk Score.

### 2.3 Definition of RCII and Other Metabolic Parameters

RC was calculated by subtracting the sum of HDL‐C and low‐density lipoprotein cholesterol (LDL‐C) from total cholesterol (TC). Subsequently, the RCII was derived by multiplying RC by hsCRP (mg/L) and dividing by 10, as previously described by Chen et al [27]. Additionally, TyG index was defined as the natural logarithm of the product of fasting glucose and triglyceride levels divided by two. CRI was calculated as the ratio of LDL‐C to HDL‐C. Furthermore, UHR was obtained by dividing serum uric acid concentration by HDL‐C concentration. The CH was then calculated by dividing hsCRP by HDL‐C and multiplying the result by 1000. Finally, the AIP was defined as the base‐10 logarithm of the molar ratio of triglycerides to HDL‐C [28].

### 2.4 Covariate

Based on prior research and expert recommendations, potential confounders and effect modifiers at baseline were identified, including age, sex (male or female), alcohol consumption, smoking status, race (in NHANES), and education level (less than high school, high school, or college). Clinical markers such as serum uric acid, creatinine, blood lipids, and glucose were measured in the laboratory.

Cardiovascular disease (CVD, specifically heart disease) was assessed via a standardized questionnaire asking whether participants had ever been diagnosed by a physician [29]. Hypertension was defined by any of the following criteria: self‐reported physician diagnosis, measured systolic blood pressure ≥ 140 mmHg, diastolic blood pressure ≥ 90 mmHg, or current use of antihypertensive medications [30]. Diabetes mellitus was diagnosed based on the presence of one or more of the following: physician‐diagnosed diabetes, hemoglobin A1c (HbA1c) ≥ 6.5%, fasting plasma glucose (FPG) ≥ 7.0 mmol/L, random blood glucose ≥ 11.1 mmol/L, 2‐h post‐load glucose during an oral glucose tolerance test (OGTT) ≥ 11.1 mmol/L, or current use of insulin or other antidiabetic drugs [31]. Hyperlipidemia was defined by meeting any of the following: triglycerides (TG) ≥ 150 mg/dL; abnormal cholesterol profile, including TC ≥ 200 mg/dL, LDL‐C ≥ 130 mg/dL, or reduced HDL‐C levels (< 40 mg/dL for males, < 50 mg/dL for females); or current use of lipid‐lowering medication [31]. Alcohol consumption was categorized as yes or no, and smoking status was classified as current/former smoker versus never smoker [32, 33].

### 2.5 Statistical Analysis

Sample weights were incorporated to account for the complex, multistage probability sampling design of NHANES. In accordance with NHANES analytic guidelines, the selection of appropriate weights was based on the variables most representative of the targeted subpopulation, ensuring valid population‐level estimates [34]. Continuous variables with a normal distribution were presented as means with standard errors, whereas those with a non‐normal distribution were expressed as median and interquartile range (IQR). Categorical variables were summarized as counts and percentages. Baseline group comparisons were performed using the chi‐square test for categorical variables, one‐way analysis of variance (ANOVA) for normally distributed continuous variables, and the Kruskal–Wallis rank‐sum test for non‐normally distributed variables [35].

Receiver operating characteristic (ROC) curves and corresponding area under the curve (AUC) values were used to evaluate the discriminatory performance of RC, RCII, TyG, CRI, CH, UHR, and AIP for identifying all‐cause mortality in both the NHANES and CHARLS cohorts. The parameter with the highest AUC was selected for further analysis using restricted cubic spline (RCS) models to explore potential nonlinear associations, with knots placed at the 10th, 50th, and 90th percentiles of its distribution. Cox proportional hazards regression models were employed to estimate hazard ratios (HRs) and 95% confidence intervals (CIs) for the association between RCII and all‐cause mortality in the overall population and in participants with CKM stages 0–3. To balance between avoiding overadjustment and maximizing the use of relevant covariates, two models were constructed. Model 1 was adjusted for age, sex, education, and smoking status. Model 2 included additional adjustments for alcohol consumption, uric acid, creatinine, CVD, diabetes, dyslipidemia, glucose, hypertension, LDL‐C, and races in NHANES; and for BMI, uric acid, creatinine, heart disease, diabetes, dyslipidemia, glucose, hypertension, TC, and LDL‐C in CHARLS. In the CKM stage 0–3 subgroup, CVD or heart disease was excluded from the covariates due to its overlap with CKM syndrome.

Stratified analyses were conducted by age, sex and BMI shown as Forrest plots. All statistical analyses were performed in R (version 4.2.1). A two‐sided *P*‐value of < 0.05 was considered statistically significant [36].

## 3 Results

### 3.1 Study Participants and Baseline Characteristics

This cross‐national analysis included data from two population‐based cohorts: 9476 participants from the NHANES and 7840 respondents from the CHARLS (Figure 1 and Table 1). In the NHANES cohort, deceased participants (*n* = 1931) were significantly older than survivors (65.83 ± 0.50 vs. 43.33 ± 0.31 years, *p* < 0.0001) and more likely to be male (52.71% vs. 48.40%, *p* = 0.006). They had significantly higher levels of glucose, serum creatinine, and uric acid (all *p* < 0.0001). The prevalence of diabetes mellitus, CVD, hypertension, and hyperlipidemia was markedly higher in the death group (all *p* < 0.0001). In addition, current or former smoking and alcohol abstinence were more common among those who died (both *p* < 0.0001). Educational attainment and racial distribution also differed significantly between groups (*p* < 0.0001). The average follow‐up duration was shorter in the mortality group (7.69 ± 0.15 vs. 13.32 ± 0.08 years, *p* < 0.0001).

**TABLE 1:** Baseline characteristics of participants in NHANES and CHARLS.

	NHANES			CHARLS		
	*Alive* (*n* = 7, 545)	*Death* (*n* = 1, 931)	*p* value	*Alive* (*n* = 7, 637)	*Death* (*n* = 203)	*p* value
Age (years)	43.330 (0.312)	65.833 (0.498)	< 0.0001	57.80 (8.79)	69.20 (9.86)	< 0.001
Sex (Male %)	3669 (48.399)	1084 (52.710)	0.006	3400 (44.52)	116 (57.14)	< 0.001
BMI (kg/m^2^)	28.448 (0.098)	28.510 (0.195)	0.771	23.72 (3.84)	21.99 (4.14)	< 0.001
Glucose (mg/dL)	100.699 (0.344)	113.288 (1.027)	< 0.0001	108.89 (31.03)	119.25 (61.86)	< 0.001
Creatinine (mg/dL)	0.869 (0.003)	1.053 (0.019)	< 0.0001	0.77 (0.18)	0.86 (0.42)	< 0.001
Uric acid (mg/dL)	5.438 (0.019)	5.812 (0.048)	< 0.0001	4.40 (1.22)	4.77 (1.55)	< 0.001
Races (%)			< 0.0001			
Black	1430 (10.893)	319 (10.314)				
White	3630 (70.789)	1275 (80.099)				
Others	2485 (18.317)	337 (9.588)				
Education (%)			< 0.0001			< 0.01
Less Than High School	1930 (16.047)	707 (28.260)		6802 (89.07)	194 (95.57)	
College	3842 (59.600)	717 (42.105)		126 (1.65)		
High School	1773 (24.353)	507 (29.635)		709 (9.28)	9 (4.43)	
TC (mg/dL)	196.095 (0.633)	197.327 (0.977)	0.22	194.43 (37.75)	187.02 (43.47)	0.006
HDL‐C (mg/dL)	54.038 (0.270)	54.261 (0.390)	0.611	51.05 (15.08)	51.08 (16.07)	0.98
LDL‐C (mg/dL)	117.344 (0.551)	114.714 (0.895)	0.009	117.76 (34.52)	111.28 (36.94)	0.008
RC (mg/dL)	24.713 (0.216)	28.353 (0.370)	< 0.0001	25.62 (24.44)	24.67 (21.00)	0.581
CRP (mg/dL)	0.371 (0.009)	0.631 (0.035)	< 0.0001	2.43 (6.41)	8.80 (18.11)	< 0.001
TyG	8.589 (0.010)	8.839 (0.014)	< 0.0001	8.68 (0.64)	8.71 (0.67)	0.566
CRI	2.350 (0.016)	2.313 (0.027)	0.234	2.47 (0.94)	2.36 (0.98)	0.097
AIP	−0.041 (0.005)	0.027 (0.007)	< 0.0001	0.00 (0.33)	‐0.02 (0.30)	0.621
RCII	9.840 (0.255)	18.148 (1.345)	< 0.0001	6.44 (27.03)	19.40 (47.19)	< 0.001
CH	7.721 (0.186)	13.593 (0.862)	< 0.0001	54.80 (152.27)	213.20 (523.58)	< 0.001
UHR	0.111 (0.001)	0.120 (0.001)	< 0.0001	0.10 (0.04)	0.10 (0.06)	0.008
Diabetes Mellitus (%)	1009 (9.764)	626 (30.843)	< 0.0001	416 (5.45)	19 (9.36)	0.02
CVD/heart disease (%)			< 0.0001			< 0.0001
No	7068 (95.164)	1340 (70.647)		6782 (88.80)	159 (78.33)	
Yes	477 (4.836)	591 (29.353)		855 (11.20)	44 (21.67)	
Hypertension (%)			< 0.0001			< 0.0001
No	4897 (69.174)	591 (33.929)		5707 (74.73)	114 (56.16)	
Yes	2648 (30.826)	1340 (66.071)		1930 (25.27)	89 (43.84)	
Hyperlipidemia (%)			< 0.0001			0.86
No	2072 (29.361)	351 (17.885)		6874 (90.01)	184 (90.64)	
Yes	5473 (70.639)	1580 (82.115)		763 (9.99)	19 (9.36)	
Smoke status (%)			< 0.0001			< 0.0001
Current/former	3377 (45.881)	1168 (61.888)		2831 (37.07)	112 (55.17)	
Never	4168 54.119)	763 (38.112)		4806 (62.93)	91 (44.83)	
Alcohol drink (%)			< 0.0001			0.09
No	2247 (25.347)	978 (46.063)		5112 (66.94)	148 (72.91)	
Yes	5298 (74.653)	953 (53.937)		2525 (33.06)	55 (27.09)	
Follow up time (years)	13.318 (0.079)	7.694 (0.153)	< 0.0001	8.99 (0.08)	2.88 (2.76)	< 0.001

Abbreviations: AIP, atherogenic index of plasma; BMI, body mass index; CH, hs‐CRP/HDL‐C ratio; CRI, Castelli risk index; CRP, C‐reactive protein; CVD, cardiovascular disease; HDL, high‐density lipoprotein; LDL, low‐density lipoprotein; RC, remnant cholesterol; RCII, remnant cholesterol inflammatory index; TC, total cholesterol; TyG, triglyceride‐glucose index; UHR, uric acid to high‐density lipoprotein cholesterol ratio.

In the CHARLS cohort, deceased participants (*n* = 203) were older (69.20 ± 9.86 vs. 57.80 ± 8.79 years, *p* < 0.001), more likely to be male (57.14% vs. 44.52%, *p* < 0.001), and had lower BMI (21.99 ± 4.14 vs. 23.72 ± 3.84 kg/m², *p* < 0.001). They also exhibited significantly higher levels of glucose, creatinine, and uric acid (all *p* < 0.001), and were more likely to have diabetes, heart disease, and hypertension (all *p* < 0.05). Smoking and educational attainment differed significantly by survival status (*p* < 0.0001 and *p* < 0.01, respectively). The mean follow‐up time was significantly shorter among those who died (2.88 ± 2.76 vs. 8.99 ± 0.08 years, *p* < 0.001).

Regarding metabolic markers, levels of RC, RCII, TyG, CH, UHR, and AIP were significantly higher in deceased participants in the NHANES cohort (all *p* < 0.0001), while CRI showed no significant difference. In the CHARLS cohort, RCII, CH, and UHR were significantly elevated among those who died (*p* < 0.01), while no significant differences were observed in RC, TyG, AIP, or CRI.

### 3.2 ROC Curves Analyses

**FIGURE 2:**
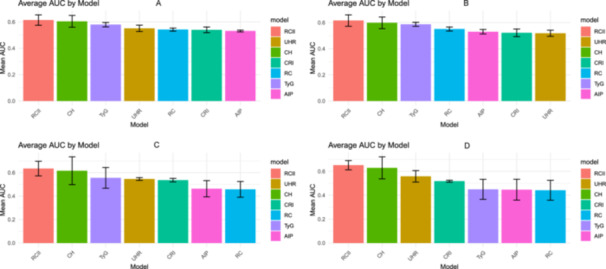
ROC curves of different metabolic parameters for all‐cause mortality predicting. (A) General population from NHANES; (B) Individuals of CKM stage 0–3 from NHANES; (C) General population from CHARLS; (D) Individuals of CKM stage 0–3 from CHARLS.

A total of 8302 and 6814 individuals with CKM stages 0–3 were identified from the NHANES and CHARLS cohorts, respectively. Time‐dependent ROC (Figure 2) and AUC curves (Supporting Figure 1A–D) were conducted separately in four subgroups: the overall population (Figure 2A,C) and individuals with CKM stages 0–3 (Figure 2B,D) in both cohorts. Among the seven metabolic parameters evaluated (RC, RCII, TyG, CRI, CH, UHR, and AIP), RCII consistently demonstrated the highest discriminatory power for predicting all‐cause mortality across all subgroups. In each of the four ROC curves—NHANES total population, NHANES CKM stages 0–3, CHARLS total population, and CHARLS CKM stages 0–3—the AUC for RCII exceeded 0.60, indicating moderate predictive performance and superiority over the other metabolic markers.

### 3.3 Results of RCS Analyses

**FIGURE 3:**
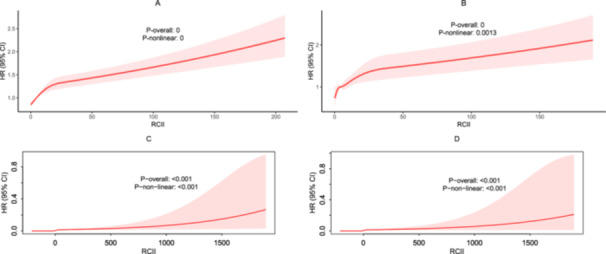
RCS curves of RCII to predict all‐cause mortality. (A) General population from NHANES; (B) Individuals of CKM stage 0–3 from NHANES; (C) General population from CHARLS; (D) Individuals of CKM stage 0–3 from CHARLS.

RCS models with three knots placed at the 10th, 50th, and 90th percentiles of the RCII distribution were fitted to visualize the dose‑response relationship in Figure 3. In both cohorts, the smoothed curves revealed an almost linear, monotonic rise in the risk of all‑cause mortality as RCII increased. For the overall populations (Figure 3A,C), likelihood‑ratio tests for non‑linearity were statistically significant (*p*  <  0.05), and an analogous pattern was observed in participants with CKM stages 0–3 (Figure 3B,D), where RCII again displayed a steadily increasing association with mortality and the non‑linearity tests were likewise significant (both *p*  <  0.05).

### 3.4 Associations Between RCII and All‐Cause Mortality in General Population and CKM Stages 0–3 in NHANES and CHARLS

Multivariable cox regression analyses were performed to examine the associations between RCII and all‐cause mortality (Table 2). Across both the NHANES and CHARLS cohorts, higher levels of the RCII were consistently associated with an increased risk of all‐cause mortality. When analyzed as a continuous variable, RCII showed a significant positive association with mortality in all models (both *p* < 0.001). In categorical analyses based on tertiles, participants in the highest RCII tertile (Q3) had substantially elevated risks of death compared to those in the lowest tertile (Q1), with fully adjusted HRs of 1.48 (95% CI: 1.28–1.71) in NHANES and 2.63 (95% CI: 1.77–3.90) in CHARLS (both *p* < 0.001). A modest but significant increase in risk was also observed in the middle tertile (Q2), with adjusted HRs of 1.18 (95% CI: 1.00–1.39) in NHANES and 1.63 (95% CI: 1.09–2.43) in CHARLS. In subgroup analyses limited to individuals diagnosed with CKM stages 0–3, the association remained robust. Participants in Q3 had HRs of 1.54 (95% CI: 1.29–1.83) in NHANES and 3.15 (95% CI: 1.97–5.03) in CHARLS, while those in Q2 also exhibited significantly increased risks. These findings suggest that elevated RCII is an independent predictor of all‐cause mortality across general populations and CKM stages 0–3.

**TABLE 2:** Risk classification of all‐cause mortality based on RCII by multiple Cox regression analysis in NHANES and CHARLS.

	NHANES			CHARLS		
	Model 0	Model 1^a^	Model 2^b^	Model 0	Model 1^a^	Model 2^b^
**All population**						
RCII	1.00 (1.00,1.01)***	1.00 (1.00,1.00)***	1.00 (1.00,1.00)***	1.00 (1.00,1.00)***	1.00 (1.00,1.00)***	1.00 (1.00,1.00)***
Q1	ref	ref	ref	ref	ref	ref
Q2	1.92 (1.63,2.27)***	1.20 (1.01,1.43)**	1.18 (1.00,1.39)*	1.60 (1.08,2.38)*	1.57 (1.06, 2.33)*	1.63 (1.09, 2.43)*
Q3	2.41 (2.09,2.77)***	1.59 (1.39,1.82)***	1.48 (1.28,1.71)***	2.51 (1.74,3.62)***	2.40 (1.66, 3.47)***	2.63 (1.77, 3.90)***
**CKM stage** 0–3						
RCII	1.00 (1.00,1.01)***	1.00 (1.00,1.00)***	1.00 (1.00,1.00)***	1.00 (1.00,1.00)***	1.00 (1.00,1.00)***	1.00 (1.00,1.00)***
Q1	ref	ref	ref	ref	ref	ref
Q2	1.94 (1.59,2.36)***	1.21 (1.00,1.48)*	1.24 (1.02,1.51)*	1.90 (1.19,3.03)**	1.90 (1.19, 3.03)**	2.02 (1.26, 3.25)**
Q3	2.41 (2.05,2.84)***	1.53 (1.31,1.79)***	1.54 (1.29,1.83)***	2.74 (1.76,4.26)***	2.67 (1.71, 4.16)***	3.15 (1.97, 5.03)***

*Note*: NHANES: ^a^Model 1 adjusted for age, sex, education, and smoke. ^b^Model 2 adjusted for age, sex, education, smoke, alcohol drink, uric acid, creatinine, CVD, diabetes, hyperlipidemia, glucose, hypertension, LDL and races (in CKM stage 0‐3, CVD as covariate was removed in model 2). CHARLS: ^a^Model 1 adjusted for age, sex, education, and smoke. ^b^Model 2 adjusted for age, sex, education, smoke, BMI, uric acid, creatinine, heart disease, diabetes, hyperlipidemia, glucose, hypertension, TC and LDL (in CKM stage 0‐3, heart disease as covariate was removed in model 2).

* *p* < 0.05

** *p* < 0.01

*** *p* < 0.001.

### 3.5 Subgroup Analyses

**FIGURE 4:**
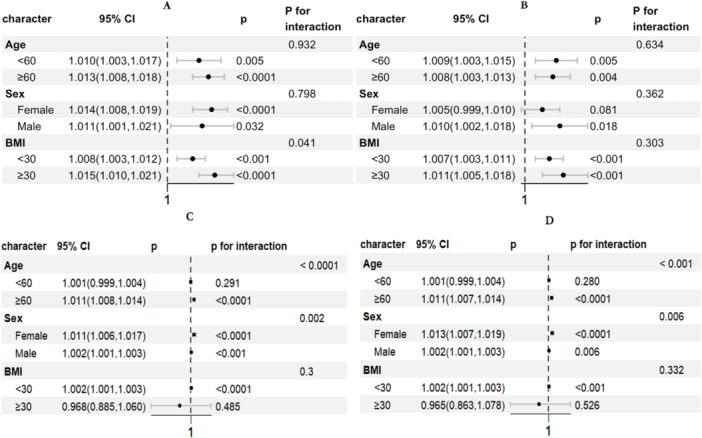
Forrest plot of subgroups analysis. (A) General population from NHANES; (B) Individuals of CKM stage 0–3 from NHANES; (C) General population from CHARLS; (D) Individuals of CKM stage 0–3 from CHARLS.

Subgroup analyses (Figure 4) demonstrated that RCII was associated with all‐cause mortality in specific populations. In Figure 4A,B, based on the NHANES dataset, higher RCII levels were significantly associated with increased mortality among males with CKM stages 0–3. This association remained significant across subgroups stratified by age (< 60 and ≥ 60 years) and BMI ( < 30 and ≥ 30 kg/m^2^), both in the general population and among individuals with CKM stages 0–3. In Figure 4C,D, using the CHARLS dataset, elevated RCII was significantly associated with increased mortality in participants aged ≥ 60 years and those with BMI < 30 kg/m^2^ These associations were consistent across both sexes, in the general population as well as in CKM stages 0–3.

### 3.6 Cause Specific Mortality Comparison

In NHANES, cause‐specific mortality analyses were further conducted to examine the associations between RCII and cardiovascular as well as non‐cardiovascular mortality (Table S1). When analyzed as a continuous variable, RCII was significantly associated with both cardiovascular and non‐cardiovascular mortality in the overall population and among individuals with CKM stages 0–3 across all models (all *p* < 0.001).

In categorical analyses, participants in the highest RCII tertile (Q3) exhibited a significantly increased risk of both cardiovascular and non‐cardiovascular mortality compared with those in the lowest tertile (Q1), after full multivariable adjustment. In contrast, associations for the middle tertile (Q2) were attenuated and no longer statistically significant in most fully adjusted models.

## 4 Discussion

In this large, cross‐national study, we found that RCII was a strong and independent predictor of all‐cause mortality across both NHANES and CHARLS cohorts. The present study extends existing literature in several important ways. First, instead of evaluating RCII in isolation, we directly compared its prognostic performance with multiple widely used metabolic biomarkers under a unified analytical framework. Second, we focused specifically on individuals with early CKM stages (0–3), a population that has been largely underrepresented in prior RCII‐related mortality studies. Finally, the consistency of findings across two nationally representative cohorts enhances the generalizability of these observations.

RC and hs‐CRP have been extensively studied as independent predictors of morbidity and mortality across a range of clinical populations. RC, as the cholesterol component of triglyceride‐rich lipoproteins, is highly atherogenic and has been linked to cardiovascular and all‐cause mortality independent of traditional lipid measures [37, 38, 39, 40, 41, 42]. Similarly, hs‐CRP is a well‐established marker of systemic inflammation and has demonstrated consistent associations with adverse outcomes across diverse clinical populations. Together, these findings support the relevance of lipid‐related and inflammatory processes to long‐term mortality risk [43, 44, 45, 46].

The superior prognostic performance of RCII observed in the present study may reflect its ability to capture overlapping metabolic and inflammatory stress that is not adequately represented by single‐domain markers. In both cohorts, RCII demonstrated a stable and monotonic association with mortality, suggesting that individuals with concurrent elevations in remnant cholesterol and systemic inflammation represent a particularly vulnerable subgroup. In cause‐specific analyses conducted in NHANES, RCII showed comparable associations with cardiovascular and non‐cardiovascular mortality, both in the overall population and among individuals with CKM stages 0–3 (Table S1). The similarity in effect estimates suggests that RCII does not function as a cardiovascular‐specific marker, but rather reflects a systemic metabolic–inflammatory burden that contributes to mortality across multiple disease pathways. This combined signal may indicate sustained metabolic burden over time rather than transient abnormalities in lipid or inflammatory parameters alone [47, 48]. This reciprocal relationship suggests that concurrent elevations in RC and hs‐CRP may reflect a high‐risk metabolic‐inflammatory phenotype. To better capture this interaction, the RCII was proposed as a composite index that integrates residual cholesterol burden and systemic inflammation. Though still a relatively novel biomarker, RCII has shown promising predictive value in studies on ischemic stroke, and adverse cardiovascular outcomes [2, 27]. However, its role in predicting all‐cause mortality in general populations remains largely unexplored.

In our study, RCII was consistently associated with all‐cause mortality across two nationally representative cohorts spanning Western and Asian populations. Compared with other metabolic indices, RCII demonstrated superior discriminatory performance based on ROC analyses. Restricted cubic spline models further showed a monotonic, near‐linear increase in mortality risk with rising RCII levels, supporting its relevance as a continuous risk indicator [49, 50].

A particularly novel aspect of our findings is the strong and consistent association between RCII and mortality across CKM stages 0–3. The CKM framework, recently proposed by the American Heart Association, emphasizes the continuum of metabolic, cardiovascular, and kidney dysfunction, and the need for early‐stage risk identification and prevention [51]. Our results show that RCII predicts mortality even in early CKM stages (0–3), indicating that RCII may capture early, subclinical processes of vascular and metabolic injury. Rather than delineating isolated biological pathways, our findings suggest that RCII captures the cumulative metabolic–inflammatory burden reflected in real‐world populations. The near‐linear association observed between RCII and adverse outcomes indicates that incremental elevations in this composite index correspond to progressively higher systemic risk. This pattern supports the concept that lipid dysregulation and inflammation operate synergistically throughout CKM development, particularly before overt clinical disease becomes apparent. The consistent associations observed in both the U.S. and Chinese populations support the external validity of RCII and its potential applicability across diverse ethnic and healthcare settings. Moreover, traditional CKM staging relies heavily on diagnosed disease thresholds such as reduced eGFR or established CVD, which may miss individuals with ongoing subclinical damage [52]. RCII could thus complement existing CKM staging systems by identifying high‐risk individuals earlier and more precisely, enabling timely intervention.

In subgroup analyses, the associations between RCII and all‐cause mortality were attenuated or non‐significant in certain subgroups, including males with CKM and younger individuals. These findings should be interpreted with caution. First, individuals at earlier CKM stages and younger ages generally have a lower baseline risk and fewer outcome events, which may reduce statistical power to detect significant associations. Second, sex‐ and age‐related differences in lipid metabolism and inflammatory responses may modify the impact of RCII on mortality risk, particularly in early disease stages where compensatory mechanisms remain intact. Third, RCII may capture cumulative metabolic–inflammatory burden, the adverse effects of which become more evident with aging and disease progression. From a clinical perspective, these findings suggest that RCII may have greater utility for risk stratification in populations with higher baseline risk, such as older individuals or those approaching advanced CKM stages, whereas its prognostic value in younger or lower‐risk subgroups may be more limited.

Taken together, RCII showed robust associations with all‐cause mortality and consistently outperformed several commonly used metabolic indices. These findings support its potential role in early risk stratification within the CKM framework. At the same time, established cardiovascular risk scores are primarily designed for long‐term cardiovascular endpoints and were not the focus of the present analysis. Whether incorporation of RCII into existing risk models could further improve risk discrimination warrants investigation in future studies.

Methodologically, this study applied a comparative framework to evaluate the prognostic performance of RCII against multiple metabolic markers using complementary analytical approaches. The inclusion of two nationally representative cohorts from distinct geographic settings enhances the robustness and external validity of the findings.

Nevertheless, this study has several limitations. First, RCII was measured only at baseline, and changes over time were not captured. Future longitudinal studies are warranted to determine whether temporal fluctuations in RCII influence mortality risk. Second, cause‐specific mortality data were unavailable in the CHARLS cohort and thus were not analyzed in either dataset, limiting assessment of RCII's association with specific causes of death and potentially constraining generalizability. Third, an additional methodological consideration is the use of different inflammatory biomarkers across cohorts. NHANES measured conventional CRP, whereas CHARLS assessed hs‐CRP. Although hs‐CRP provides greater sensitivity at lower concentration ranges, both assays quantify the same underlying inflammatory protein and have been shown to be highly correlated in population‐based studies. Importantly, our analyses focused on relative risk estimation and within‐cohort ranking of RCII rather than absolute CRP thresholds. Moreover, RCII was standardized and categorized within each cohort, which is expected to mitigate potential bias arising from differences in assay sensitivity. Therefore, while differences in CRP measurement may limit direct comparison of absolute RCII values across cohorts, they are unlikely to substantially affect the observed associations with mortality. Finally, cause‐specific mortality analyses were limited to the NHANES cohort, as the CHARLS dataset does not distinguish cardiovascular from non‐cardiovascular causes of death. This difference reflects heterogeneity in mortality ascertainment across cohorts rather than analytical design, and should be considered when interpreting cross‐cohort comparisons.

## 5 Conclusion

This study demonstrated that RCII is a strong and independent predictor of all‐cause mortality across general and CKM stage 0–3 populations in both U.S. and Chinese cohorts. Compared to other commonly used metabolic indices, RCII consistently showed superior discriminatory performance and a stable dose–response relationship with mortality risk. Its predictive value remained robust across most demographic and clinical subgroups, underscoring its potential utility as a practical tool for early risk stratification in diverse populations. These findings highlight the importance of integrating lipid‐related and inflammatory pathways in cardiometabolic risk assessment and suggest that RCII may serve as a valuable marker for mortality surveillance and prevention strategies in the context of CKM syndrome.

## Author Contributions

Chen‐Zhang Ou and Zhong‐Qiang Liu conceived and designed the study, acquired the data and drafted the manuscript. Zhong‐Qiang Liu analyzed the data. Yu‐Jun Xiong and Shiqin Chen contributed to the interpretation of the results and critical revision of the manuscript for important intellectual content. Tian Lv developed the software and provided technical support. Jingjing Lou had the primary responsibility for final content. All authors have read and approved the final manuscript.

## Funding

The authors have nothing to report.

## Ethics Statement

The study was conducted in accordance with the Declaration of Helsinki (as revised in 2013). For NHANES, informed consent was obtained from all participants, and the study protocol was approved by the National Center for Health Statistics (NCHS) Ethics Review Board (Protocol #2011–17). Since all NHANES data are publicly available, additional approval from a medical ethics committee was not required. The CHARLS study also adhered to the ethical principles of the Declaration of Helsinki and received approval from the Institutional Review Board of Peking University (IRB00001052‐11015). Furthermore, the research involving human participants was approved by the Ethics Committee of Peking University. Written informed consent was obtained from all participants prior to their involvement in the study.

## Conflicts of Interest

The authors declare no conflicts of interest.

## Supporting information

Supporting File 1

Supporting File 2

## Data Availability

The datasets used and/or analyzed in this research are publicly accessible at http://charls.pku.edu.cn/en and https://www.cdc.gov/nchs/nhanes/.
