# Supplementary figures and images for: Remnant Cholesterol Inflammatory Index and Its Association With All‐Cause Mortality Among General Population and Individuals With Cardiovascular–Kidney–Metabolic Syndrome Stages 0–3: Evidence From Two Nationwide Studies
Source: Clin Cardiol. 2026 Apr 13;49(4):e70297. doi: 10.1002/clc.70297 (PMC13074494; doi:10.1002/clc.70297)

A

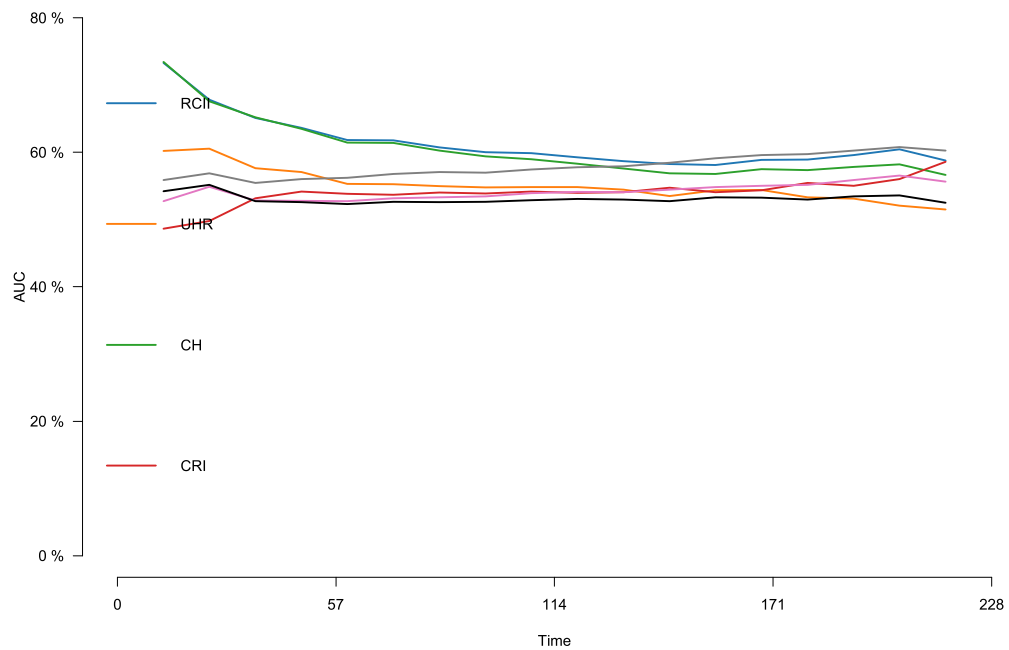

B

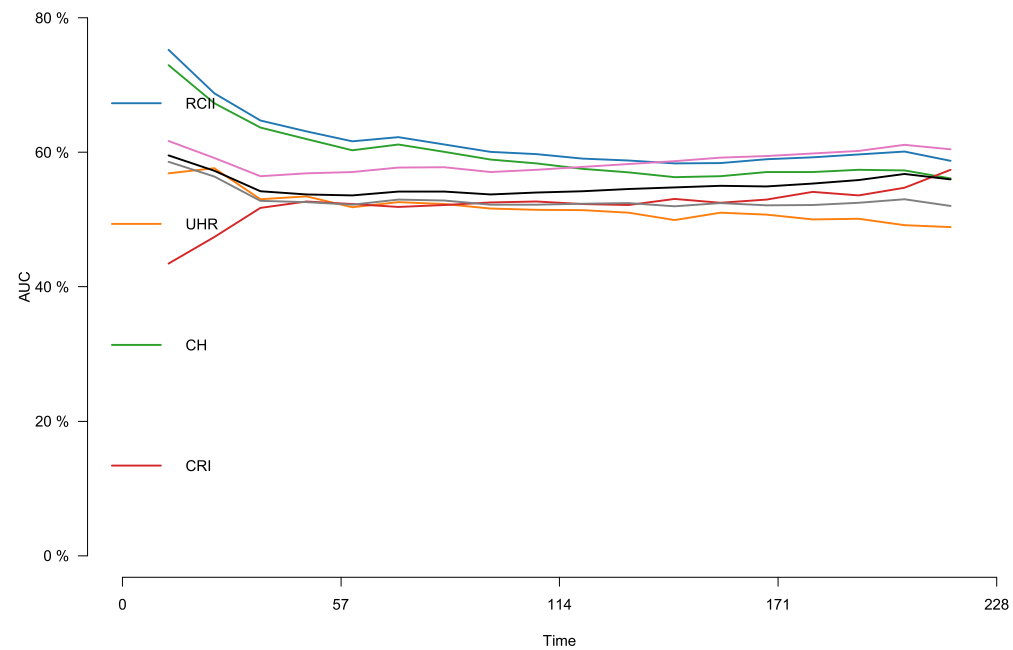

C

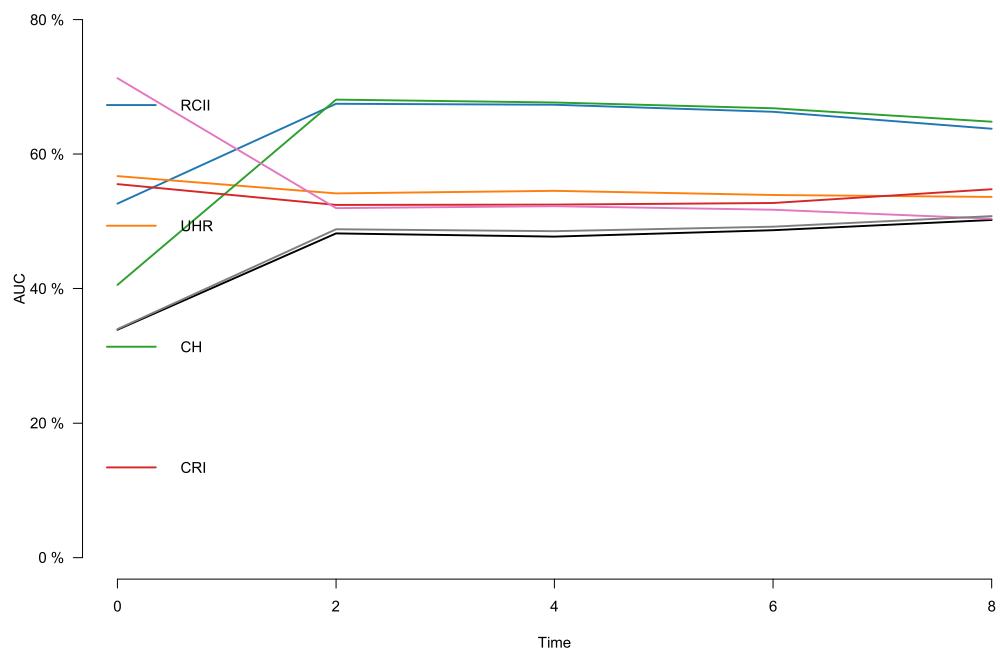

D

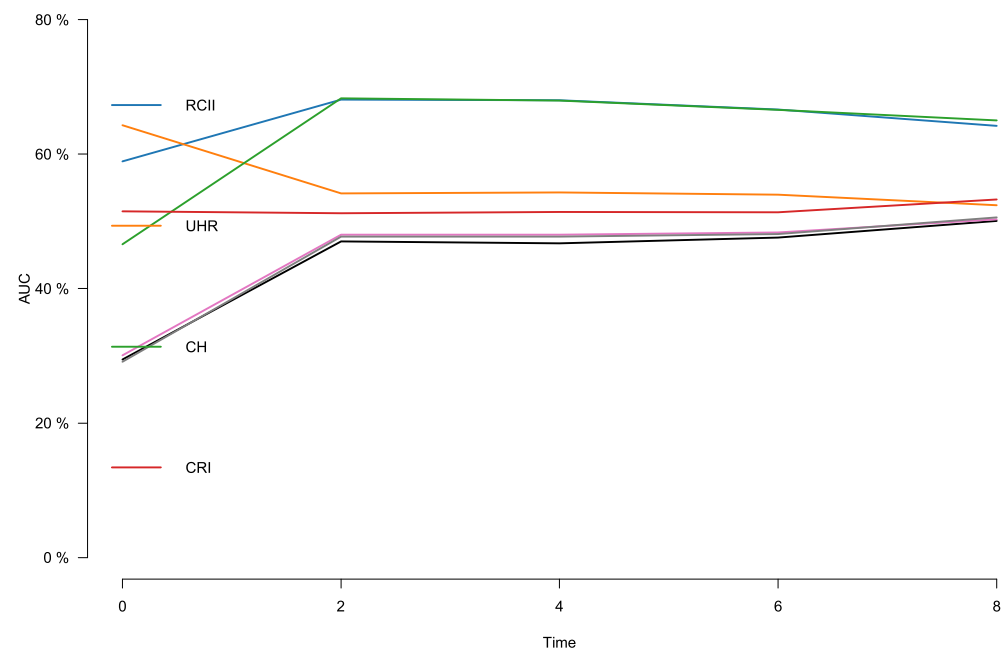

Supplement: Supplementary file 1 — Supporting File 1 [file CLC-49-e70297-s001.pdf]
