# Supplementary material for: Remnant Cholesterol Inflammatory Index and Its Association With All‐Cause Mortality Among General Population and Individuals With Cardiovascular–Kidney–Metabolic Syndrome Stages 0–3: Evidence From Two Nationwide Studies
Source: Clin Cardiol. 2026 Apr 13;49(4):e70297. doi: 10.1002/clc.70297 (PMC13074494; doi:10.1002/clc.70297)
Supplement: Supplementary file 2 — Supporting File 2 [file CLC-49-e70297-s002.docx]

Supplementary Table 1 Risk classification of Specific Cause Mortality based on RCII by Multiple Cox Regression analysis in NHANES.

|  | CVD mortality | | | Non-CVD mortality | | |
| --- | --- | --- | --- | --- | --- | --- |
|  | Model 0 | Model 1^a^ | Model 2^b^ | Model 0 | Model 1^a^ | Model 2^b^ |
| All population |  |  |  |  |  |  |
| RCII | 1.01(1.00,1.01) *** | 1.00(1.00,1.01) *** | 1.00(1.00,1.01) *** | 1.00(1.00,1.01) *** | 1.00(1.00,1.01) *** | 1.00(1.00,1.01) *** |
| Q1 | ref | ref | ref | ref | ref | ref |
| Q2 | 2.00(1.49,2.69) *** | 1.22(0.90,1.64) | 1.19(0.90,1.58) | 1.94(1.62,2.32) *** | 1.19(0.98,1.44) | 1.19(0.98,1.45) |
| Q3 | 2.57(2.07,3.18) *** | 1.73(1.34,2.22) *** | 1.52(1.19,1.94) *** | 2.45(2.03,2.96) *** | 1.61(1.36,1.90) *** | 1.57(1.30,1.89) *** |
| CKM stage 0-3 |  |  |  |  |  |  |
| RCII | 1.01(1.00,1.01) *** | 1.00(1.00,1.01) *** | 1.00(1.00,1.01) *** | 1.00(1.00,1.01) *** | 1.00(1.00,1.01) *** | 1.00(1.00,1.01) *** |
| Q1 | ref | ref | ref | ref | ref | ref |
| Q2 | 2.10(1.47,3.01) *** | 1.28(0.89,1.84) | 1.26(0.87,1.83) | 1.91(1.55,2.37) *** | 1.19(0.96,1.47) | 1.22(0.99,1.52) |
| Q3 | 2.55(1.89,3.44) *** | 1.64(1.20,2.23) ** | 1.52(1.07,2.16) * | 2.42(1.95,2.99) *** | 1.50(1.23,1.84) *** | 1.55(1.24,1.94) *** |

^a^ Model 1 adjusted for age, sex, education, and smoke.

^b^ Model 2 adjusted for age, sex, education, smoke, alcohol drink, uric acid, creatinine, diabetes, hyperlipidemia, glucose, hypertension, LDL and races.
